# First-degree relatives with similar phenotypic characterisation of acute myocardial infarction: a case report and review of the literature

**DOI:** 10.1186/s12872-019-01303-4

**Published:** 2019-12-27

**Authors:** Yi-Dan Hao, Bright Eric Ohene, Shi-Wei Yang, Yu-Jie Zhou

**Affiliations:** Beijing Anzhen Hospital, Capital Medical University; Beijing Institute of Heart, Lung and Blood Vessel Disease; The Key Laboratory of Remodeling-related Cardiovascular Disease, Ministry of Education, No. 2 Anzhen Road, Chao Yang District, 100029 Beijing China

**Keywords:** Coronary artery disease, Myocardial infarction, Heterogeneities of phenotype

## Abstract

**Background:**

Genetic susceptibility to the development of coronary artery disease (CAD) and myocardial infarction (MI) is well established. However, lack of replication, and difficulty in the identification of specific genes that underlie impressive linkage peaks remain challenges at the molecular level due to the heterogeneity of phenotype and their associated genotypes. We present two cases of first-degree family members of acute myocardial infarction (AMI) having similar clinical and angiographic features of obstructive coronary lesions at same anatomic locations.

**Case presentation:**

The father presented with significant chest discomfort and loss of consciousness. The electrocardiogram (ECG) showed an acute anterior ST-segment–elevation myocardial infarction (STEMI). Coronary angiogram demonstrated a subtotal occlusion in the mid-left anterior descending (LAD) coronary artery. One week later, the son presented after an in-hospital cardiac arrest with pulseless electric activity preceded by significant chest pain and loss of consciousness. His ECG also showed an acute anterior STEMI. Catheterization revealed strikingly similar angiographic characteristics with his father: subtotal occlusion in the proximal to mid-LAD coronary artery.

**Conclusions:**

More considerations should be given to patients with similar phenotypic characterization in genetic studies of CAD/MI in the future.

## Background

Coronary artery disease (CAD) is projected to become the dominant cause of death and disability worldwide by 2020 [1]. Although reduction of traditional risk factors for CAD is associated with 30 to 40% less adverse events such as death and myocardial infarction (MI) [2], residual cardiovascular risk is a challenge due to the genetic predisposition [3–8]. In recent years, some significant progress has been made in identifying genes that are associated with susceptibility to the development of CAD/MI. However, there has been remarkable lack of replication, and difficulty in identifying genes that underlie impressive linkage peaks due to the heterogeneities of phenotype and their associations with genotype. We present two first-degree family members of acute myocardial infarction (AMI) with clinical and angiographic similarities.

## Case presentation

**Fig. 1:**
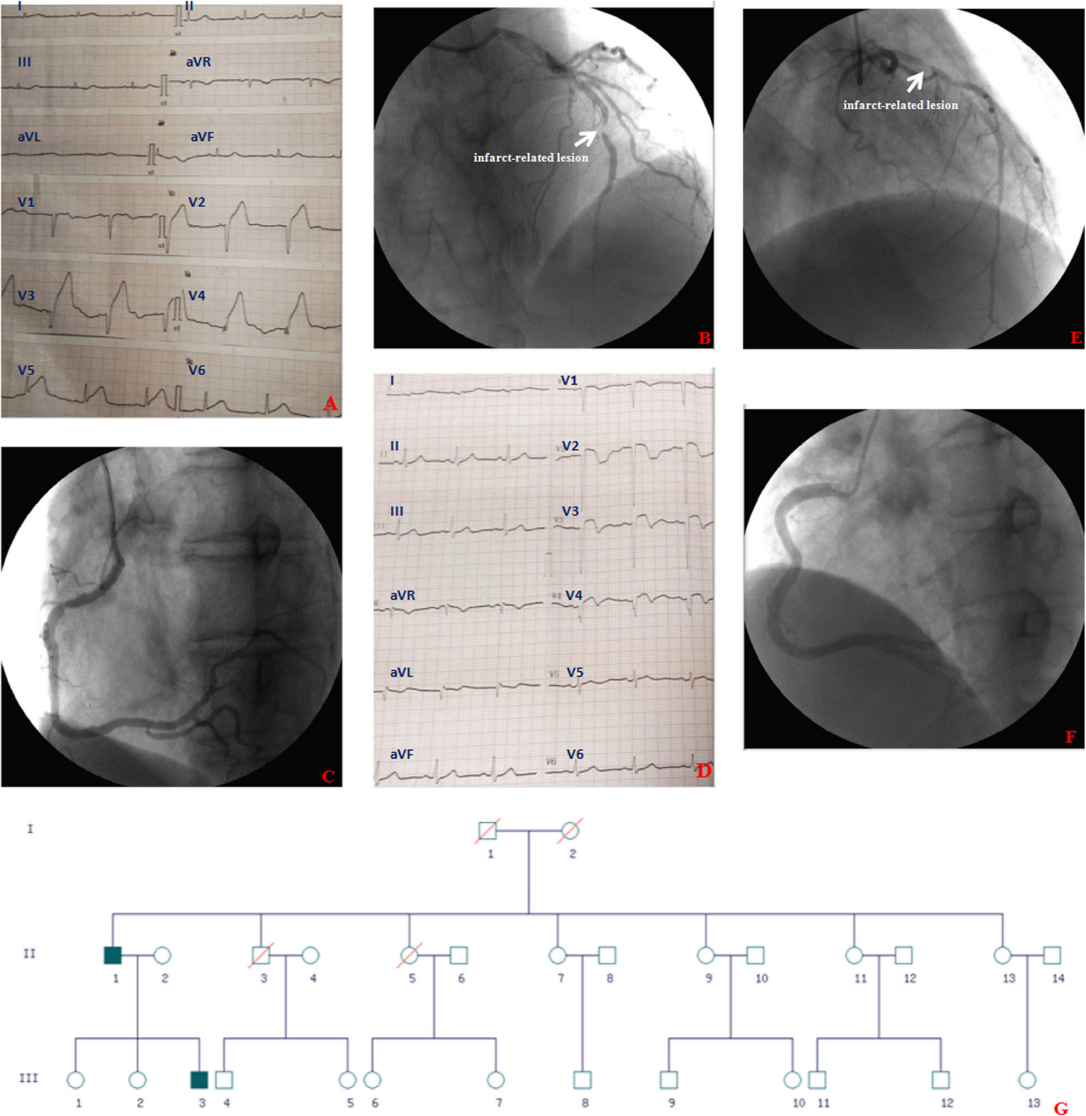
Patient’s imaging data. **a**: The father’s initial ECG demonstrating an anterior STEMI; **b**: The father’s coronary angiogram demonstrating a subtotal occlusion in middle part of LAD; **c**: The father’s coronary angiogram demonstrating moderate stenosis in the right coronary artery; **d**: The son’s initial ECG demonstrating an anterior STEMI; **e**: The son’s coronary angiogram demonstrating subtotal occlusion in the proximal to middle part of LAD; **f**: The son’s coronary angiogram demonstrating tubular disease in the right coronary artery; **g**: Pedigrees of the family

The father, a 61-year-old man with a history of heavy smoking (30 cigarettes per day for 40 years) presented with significant chest discomfort and loss of consciousness, which returned after 5 min. His initial electrocardiogram (ECG) showed an acute anterior ST-segment–elevation myocardial infarction (STEMI; Fig. 1a). In the emergency department, the symptoms were released and partial resolution of ST-segment was noted on the ECG 1 h post-thrombolysis with reteplase. Coronary angiogram demonstrated a subtotal occlusion in the mid-left anterior descending (LAD) coronary artery (Fig. 1b and Additional file 1: Video S1) with moderate disease in the right coronary artery (Fig. 1c and Additional file 2: Video S2). He successfully underwent percutaneous coronary intervention (PCI) and stent implantation. One week later after discharge, the son presented with AMI. The 32-year-old man, also with a history of heavy smoking (40 cigarettes per day for 10 years), presented after an in-hospital cardiac arrest with pulseless electric activity (PEA) preceded by significant chest pain and loss of consciousness. Cardiopulmonary resuscitation was started for PEA arrest with return of spontaneous circulation after 3 min. His initial ECG showed an acute anterior STEMI (Fig. 1d). The emergency catheterization revealed strikingly similar angiographic characteristics with his father: subtotal occlusion in the proximal to mid-LAD coronary artery (Fig. 1e and Additional file 3: Video S3) with tubular disease in the right coronary artery (Fig. 1f and Additional file 4: Video S4). He also successfully underwent PCI and stent implantation. Interestingly, either the father or the son had no history of hypertension, diabetes mellitus, hyperlipidemia, and other cardiovascular risk factors except smoking. They had no history of any emotional or psychiatric diseases. The patients were not taking any prescription medication and had no known allergies before admission. All laboratory values of the father and son were detailed in Table 1. All the members of the family were studied, which comprised only the two affected individuals (Fig. 1g).

**Table 1:** Laboratory Values of the Father and Son

Laboratory variables	Father	Son
Initial cTnI (ng/mL)	2.3	0.42
Peak cTnI (ng/mL)	> 50	> 50
Creatinine (umol/L)	86	45
FPG (mmol/L)	7.2	5.8
LDL-C (mmol/L)	1.92	2.54
HDL-C (mmol/L)	0.98	1.04
K+ (mmol/L)	4.2	3.9
LVEDD (mm)	48	45
LVEF (%)	46	53

*cTnI* cardiac troponin I, *FPG* fasting plasma glucose, *LDL-C* low-density lipoprotein cholesterol, *HDL-C* high-density lipoprotein cholesterol, *K*^*+*^ potassium ion**,***LVEDD* left ventricular end diastolic dimension, *LVEF* left ventricular ejection fraction

## Discussion and conclusion

Despite an extensive body of research dedicated to the genetic basis of CAD, most of the identified genes individually or in combination confer relatively small increments in risk (1.1- to 1.5-fold) and explain only a small proportion of heritability [9–11]. Also notable and insightful studies are available, some focusing on juvenile onset CAD, while others enrolled patients with maturity-onset CAD. And even though the studied subjects suffered from MI, their inclusion criteria varied considerably. Defectiveness in certain genetic clusters predispose to similar but clinical entities; some to CAD and others to MI. It is importance to draw clear distinction between clinical syndrome because the phenotype of CAD in some studies has been consistently switched with that of MI in another [2–16].

Carl Miller et al [17] first described the co-segregation of elevated cholesterol, xanthomas and premature CAD, providing some clues regarding a genetic trait of CAD/MI. Clinical and population-based studies have subsequently demonstrated that genetic factors play important roles in CAD/MI [18–27]. Twin studies estimated the heritability of CAD/MI to be approximately 50 to 60% [3–8]. Understanding the genetic basis of CHD/MI will not only help to understand the pathogenesis of the disease, but also lay the foundation for the development of precise prevention and treatment strategies [28].

However, scientific work at the molecular level thus the genetic architecture of CAD/MI has been formidable and ultra-expensive task owing to the heterogeneities of clinical presentation of CAD/MI and the elaborate pathophysiological processes underpinning both the genetics and the environmental factors and their interactions [28]. Clinically, atherosclerotic CAD comprises a broad spectrum of clinical entities that include completely asymptomatic myocardial ischemia, angina pectoris, and acute myocardial infarction (AMI) to sudden cardiac death.

It has been recognized that hemodynamic shear forces are largely responsible for the specific sites of the vasculature, either susceptible or resistant to developing atherosclerosis. Usually the deficiency in genes or genetic mutations may have some effects on atherosclerotic lesion burden, not the distribution of plaque [29]. However, the location-dependent effects of platelet endothelial cell adhesion molecule-1 (PECAM-1) on atherosclerotic lesions have been reported [30, 31]. The outcomes points to the different genetic influences of atherosclerosis, and their clinical heterogeneity of the location of culprit atherosclerotic lesions of CAD/MI may represent differently under different dynamic flow conditions [32]. These heterogeneities of phenotypic characterization, pathological etiologies of CAD/MI and the complex molecular and cellular pathogenesis of atherosclerosis contribute to the difficulties associated with the identification of genes that are important for CAD/MI [33].

Despite the clinical heterogeneity of CAD/MI as described above, the phenomenon of familial clustering of CAD/MI and collections of large pedigrees with multiple members in multiple generations provided an opportunity to perform linkage analysis and gene discovery. Careful consideration should be given to similar phenotypic characterisation of patients in genetic studies of CAD/MI in the future. On the basis of the concerns that have been reviewed, we present a rare case report of a first-degree relative of AMI with clinical and angiographic similarities.

There were limitations for this report. For example, we were unable to analyze the patients’ genetic background without the consent of the patients and their family. It still gives us valuable clues in further research.

In conclusion, more considerations should be given to patients with similar phenotypic characterization in genetic studies of CAD/MI in the future. It may facilitate replication and progress in this research arena.

## Supplementary information

**Additional file 1: Video S1**. Father’s left coronary angiogram.

**Additional file 2: Video S2**. Father’s right coronary angiogram.

**Additional file 3: Video S3**. Son’s left coronary angiogram.

**Additional file 4: Video S4**. Son’s right coronary angiogram.

## Data Availability

All information supporting the conclusions of this article is presented in the article.

## Abbreviations

AMI: Acute myocardial infarction
ApoE: Apolipoprotein E
CAD: Coronary artery disease
ECG: Electrocardiogram
LAD: Left anterior descending
LDLR: LDL receptor
MI: Myocardial infarction
PCI: Percutaneous coronary intervention
PEA: Pulseless electric activity
PECAM-1: Platelet endothelial cell adhesion molecule-1
STEMI: ST-segment–elevation myocardial infarction

## References

[1] Murray CJ, Lopez AD (1997). Alternative projections of mortality and disability by cause 1990-2020: global burden of disease study. Lancet..

[2] Wald NJ, Law MR (2003). A strategy to reduce cardiovascular disease by more than 80%. BMJ..

[3] Benedict RB (1958). Coronary heart disease in identical female twins. Am J Med.

[4] Sidd JJ, Sasahara AA, Littmann D (1966). Coronary-artery disease in identical twins. A family study. N Engl J Med.

[5] Kreulen TH, Cohn PF, Gorlin R (1975). Premature coronary artery disease in identical male twins studied by selective coronary arteriography. Catheter Cardiovasc Diagn.

[6] Segura L, Moreno R, Macaya C (2007). Coronary artery disease and percutaneous coronary intervention in a set of twins. Rev Esp Cardiol.

[7] Zdravkovic S, Wienke A, Pedersen NL, Marenberg ME, Yashin AI, De Faire U (2002). Heritability of death from coronary heart disease: a 36-year follow-up of 20 966 Swedish twins. J Intern Med.

[8] Wienke A, Holm NV, Skytthe A, Yashin AI (2001). The heritability of mortality due to heart diseases: a correlated frailty model applied to Danish twins. Twin Res.

[9] Manolio TA, Collins FS, Cox NJ (2009). Finding the missing heritability of complex diseases. Nature..

[10] Gibson G (2010). Hints of hidden heritability in GWAS. Nat Genet.

[11] Eichler EE, Flint J, Gibson G (2010). Missing heritability and strategies for finding the underlying causes of complex disease. Nat Rev Genet.

[12] Roberts R, Stewart AF (2012). Genes and coronary artery disease: where are we. J Am Coll Cardiol.

[13] Broeckel U, Hengstenberg C, Mayer B (2002). A comprehensive linkage analysis for myocardial infarction and its related risk factors. Nat Genet.

[14] Harrap SB, Zammit KS, Wong ZY (2002). Genome-wide linkage analysis of the acute coronary syndrome suggests a locus on chromosome 2. Arterioscler Thromb Vasc Biol.

[15] Wang Q, Rao S, Shen GQ (2004). Premature myocardial infarction novel susceptibility locus on chromosome 1P34-36 identified by genomewide linkage analysis. Am J Hum Genet.

[16] Helgadottir A, Manolescu A, Thorleifsson G (2004). The gene encoding 5-lipoxygenase activating protein confers risk of myocardial infarction and stroke. Nat Genet.

[17] Lusis AJ, Fogelman AM, Fonarow GC (2004). Genetic basis of atherosclerosis: part I: new genes and pathways. Circulation..

[18] THOMAS CB, COHEN BH (1955). The familial occurrence of hypertension and coronary artery disease, with observations concerning obesity and diabetes. Ann Intern Med.

[19] Rose G (1964). Familial patterns in ischaemic heart disease. Br J Prev Soc Med.

[20] Slack J, Evans KA (1966). The increased risk of death from ischaemic heart disease in first degree relatives of 121 men and 96 women with ischaemic heart disease. J Med Genet.

[21] Schildkraut JM, Myers RH, Cupples LA, Kiely DK, Kannel WB (1989). Coronary risk associated with age and sex of parental heart disease in the Framingham study. Am J Cardiol.

[22] Nora JJ, Lortscher RH, Spangler RD, Nora AH, Kimberling WJ (1980). Genetic--epidemiologic study of early-onset ischemic heart disease. Circulation..

[23] Anderson AJ, Loeffler RF, Barboriak JJ, Rimm AA (1979). Occlusive coronary artery disease and parental history of myocardial infarction. Prev Med.

[24] Christiansen MK. Early-onset Coronary Artery Disease Clinical and Hereditary Aspects. Dan Med J. 2017;64(9):B5406. 28874246

[25] Hamby RI (1981). Hereditary aspects of coronary artery disease. Am Heart J.

[26] Grech ED, Ramsdale DR, Bray CL, Faragher EB (1992). Family history as an independent risk factor of coronary artery disease. Eur Heart J.

[27] Shea S, Ottman R, Gabrieli C, Stein Z, Nichols A (1984). Family history as an independent risk factor for coronary artery disease. J Am Coll Cardiol.

[28] Dai X, Wiernek S, Evans JP, Runge MS (2016). Genetics of coronary artery disease and myocardial infarction. World J Cardiol.

[29] VanderLaan PA, Reardon CA, Getz GS (2004). Site specificity of atherosclerosis: site-selective responses to atherosclerotic modulators. Arterioscler Thromb Vasc Biol.

[30] Goel R, Schrank BR, Arora S (2008). Site-specific effects of PECAM-1 on atherosclerosis in LDL receptor-deficient mice. Arterioscler Thromb Vasc Biol.

[31] Harry BL, Sanders JM, Feaver RE (2008). Endothelial cell PECAM-1 promotes atherosclerotic lesions in areas of disturbed flow in ApoE-deficient mice. Arterioscler Thromb Vasc Biol.

[32] Cybulsky MI (2008). Morphing the topography of atherosclerosis: an unexpected role for PECAM-1. Arterioscler Thromb Vasc Biol.

[33] Luo AK, Jefferson BK, Garcia MJ, Ginsburg GS, Topol EJ (2007). Challenges in the phenotypic characterisation of patients in genetic studies of coronary artery disease. J Med Genet.

